# A Comprehensive Review of the Management of Acute Respiratory Distress Syndrome

**DOI:** 10.7759/cureus.30669

**Published:** 2022-10-25

**Authors:** Abimbola O Ajibowo, Olasunkanmi A Kolawole, Haleema Sadia, Oyovwike S Amedu, Hassan A Chaudhry, Helai Hussaini, Eloho Hambolu, Tuba Khan, Humaira Kauser, Aadil Khan

**Affiliations:** 1 Internal Medicine, Lugansk Medical University, Lugansk, UKR; 2 Internal Medicine, University of Texas School of Public Health, Houston, USA; 3 Internal Medicine, Khyber Teaching Hospital, Peshawar, PAK; 4 Medicine, Federal Medical Centre Abeokuta, Abeokuta, NGA; 5 Interdisciplinary Medicine, Independent Research Scholar, Philadelphia, USA; 6 ENT, Jamhoryat, Toronto, CAN; 7 Family Medicine, Sacred Heart Hospital, Lantoro, Abeokuta, NGA; 8 Medicine and Surgery, Ziauddin University, Karachi, PAK; 9 General Medicine, Dr. NTR University of Health Sciences, Vijayawada, IND; 10 Internal Medicine, Lala Lajpat Rai Hospital, Kanpur, IND

**Keywords:** mechanical ventilation, surfactant therapy, covid induced ards, rescue therapy ards, ards (acute respiratory distress syndrome)

## Abstract

Acute respiratory distress syndrome (ARDS) is an inflammatory process in the lungs that induces non-hydrostatic protein-rich pulmonary edema. ARDS occurs in roughly half of coronavirus disease 2019 (COVID-19) pneumonia patients, with most of them requiring intensive care. Oxygen saturation, partial pressure of the oxygen, and the fraction of the inspired oxygen are health indicators that may indicate a severe illness necessitating further investigation. As treatments have evolved, a typical pattern of ARDS has likewise evolved. In cases where mechanical ventilation is required, the use of low tidal volumes (<6 ml/kg ideal body weight) and airway pressures (plateau pressure <30 cmH_2_O) was recommended. For patients with moderate/severe ARDS (partial pressure to fractional inspired oxygen ratio <20), prone positioning was recommended for at least 16 hours per day. By contrast, high-frequency oscillation was not recommended. The use of inhaled vasodilators was recommended in patients with persistent hypoxemia despite invasive ventilation and prone position until extracorporeal membrane oxygenation (ECMO). The use of a conservative fluid management strategy was suggested for all patients. Mechanical ventilation with high positive end-expiratory pressure (PEEP) was suggested for patients with ARDS with a ratio of arterial oxygen partial pressure to fractional inspired oxygen (PF) ratios. ECMO was suggested as an adjunct to protective mechanical ventilation for patients with severe ARDS. In the absence of adequate evidence, research recommendations were made for corticosteroids and extracorporeal carbon dioxide removal. While decades of research have been conducted, treatment options for underlying pathologies remain limited, and mechanical ventilation, which removes carbon dioxide from the body, remains essential to achieving better clinical outcomes. This review aims to identify the best ARDS treatments that are currently available.

## Introduction and background

**Figure 1:**
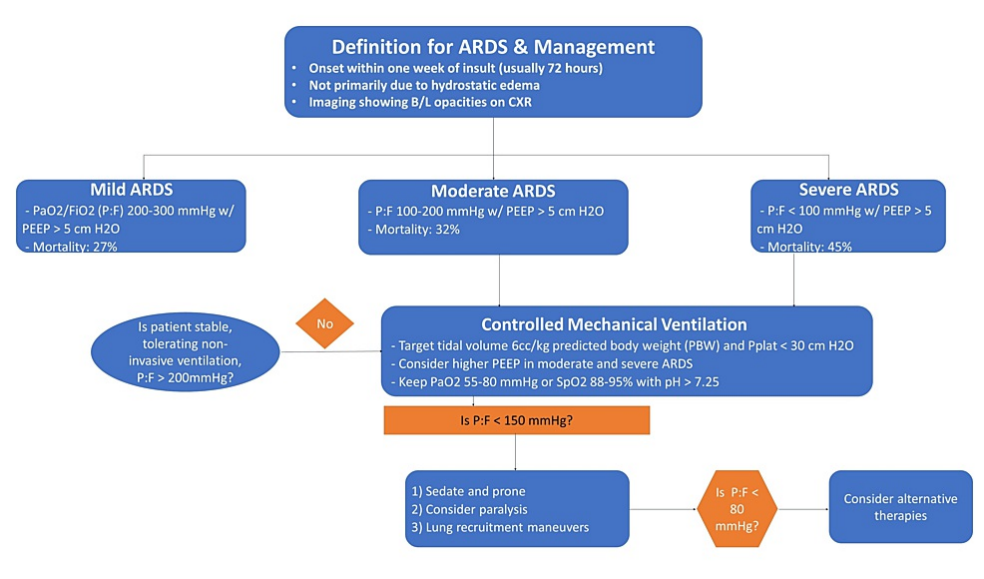
Definition and classification of ARDS ARDS: acute respiratory distress syndrome; PEEP: positive end-expiratory pressure; CXR: chest X-ray; PaO_2_: arterial pressure of oxygen; FiO_2_: fraction of inspired oxygen

Acute respiratory distress syndrome (ARDS) is an inflammatory process in the lungs that induces non-hydrostatic protein-rich pulmonary edema. The immediate consequences of the condition are profound hypoxemia, decreased lung compliance, and increased intrapulmonary shunt and dead space. The clinicopathological aspects include severe inflammatory injury to the alveolar-capillary barrier, surfactant depletion, and loss of aerated lung tissue. The European Society of Intensive Care Medicine defines ARDS as the presence of respiratory symptoms within seven days of a known clinical insult or new or worsening respiratory symptoms involving a combination of acute hypoxemia with a ratio of arterial oxygen partial pressure to fractional inspired oxygen (PF) ratios (≤300 mmHg) in a ventilated patient with positive end-expiratory pressure (PEEP) of at least 5cmH_2_O, and bilateral opacities not fully explained by heart failure or volume overload. The Berlin definition uses the PF ratio to distinguish mild ARDS (200 <PF ≤300 mmHg), moderate ARDS (100 <PF ≤200 mmHg), and severe ARDS (PF ≤100 mmHg) (Figure 1) [1].

Much information on the epidemiology of ARDS has been accrued from LUNG SAFE, an international, multicenter, prospective study conducted on over 29,000 patients in 50 countries [2,3]. ARDS accounted for 10% of ICU admissions and 23% of ventilated patients during this study. Significant physical, psychological, and cognitive sequelae, with a marked impact on quality of life, have been reported up to five years after ARDS [4,5]. One of the most important results of the LUNG SAFE study was that ARDS was not identified as such by the primary care clinician in almost 40% of cases [2]. This was particularly so for mild ARDS, in which only 51% of cases were identified [2]. All ARDS criteria were met in Only 34% of patients, suggesting a delay in adapting the treatment, mainly mechanical ventilation [2]. This is why these formal guidelines are not limited to patients presenting with severe ARDS but are intended for application to all mechanically ventilated intensive care patients. Results from the LUNG SAFE study suggest that the ventilator settings did not fully adhere to the principles of protective mechanical ventilation [2]. Plateau pressure was measured in only 40% of ARDS patients [2]. And only two-thirds of patients for whom plateau pressure was reported were receiving protective mechanical ventilation [tidal volume ≤8 mL/kg predicted body weight (PBW) and plateau pressure ≤30 cmH_2_O] [2]. Analysis of the LUNG SAFE results also shows a lack of relationship between PEEP and the PF ratio [2]. Lastly, prone positioning was used in just 8% of patients presenting with ARDS, essentially as a salvage treatment [2]. The reduction in mortality associated with ARDS over the last 20 years can be mainly attributed to a decrease in ventilator-induced lung injury (VILI). VILI is essentially related to volutrauma, closely associated with “strain” and “stress.” Lung stress corresponds to transpulmonary pressure (alveolar pressure-pleural pressure), and lung strain refers to the change in lung volume indexed to the functional residual capacity of the ARDS lung at zero PEEP. So, volutrauma corresponds to generalized excess stress and strain on the injured lung [6-8]. High-quality CT scans and physiological studies have revealed that lung lesions are unequally distributed, the injury or atelectasis coexisting with aerated alveoli of close-to-normal structure [9]. ARDS is not a disease but a syndrome defined by numerous clinical and physiological criteria. Therefore, it is not surprising that lung-protective ventilatory strategies based on underlying physiological principles effectively improve outcomes. Minimizing VILI thus generally aims to reduce volutrauma (reduction in global stress and strain). Lowering airway pressures has the dual theoretical benefit of minimizing overdistension of the aerated areas and mitigating negative hemodynamic consequences. The current guidelines are more than 20 years old, and hence there was a pressing need to update them. The main aim of these formal guidelines was to confine the topics to the best-studied fields to provide practitioners with solid guidelines with a high level of agreement between experts. Certain essential aspects of ARDS management were deliberately not addressed because there is an insufficient assessment of their effects on prognosis (respiratory rate, mechanical power, target oxygenation, pH, PaCO_2_). We also limited these guidelines to adult patients and invasive mechanical ventilation.

## Review

Pathophysiology and risk factors

ARDS occurs as a consequence of an alveolar injury due to various causes producing diffuse alveolar damage. This leads to the release of pro-inflammatory cytokines [tumor necrosis factor, interleukin (IL)-1, IL-6, IL-8], which recruit neutrophils to the lungs, where they get activated and release toxic mediators (reactive oxygen species and proteases) that damage the capillary endothelium and alveolar epithelium leading to alveolar edema [10]. This, eventually, leads to impairment of gas exchange, decreased lung compliance, and increased pulmonary arterial pressure. *Pathological Stages*

The initial exudative stage is characterized by diffuse alveolar damage. The second stage of proliferation develops after approximately 10-14 days, characterized by the resolution of pulmonary edema, the proliferation of type II alveolar cells, squamous metaplasia, interstitial infiltration by myofibroblasts, and early deposition of collagen. Some patients progress to the third stage of fibrosis, characterized by the obliteration of normal lung architecture, diffuse fibrosis, and cyst formation [11].

Management of ARDS

Currently, no specific drugs or therapies are available to treat/prevent ARDS directly. Mechanical ventilation to minimize ventilator-induced lung injury (VILI) and management of refractory hypoxemia are the keystones in the supportive management of ARDS [12]. We engage in a review of the recommended ventilator strategies, various pharmacological and nonpharmacological therapies available, and current recommendations for optimal management of patients with ARDS.

**Figure 2:**
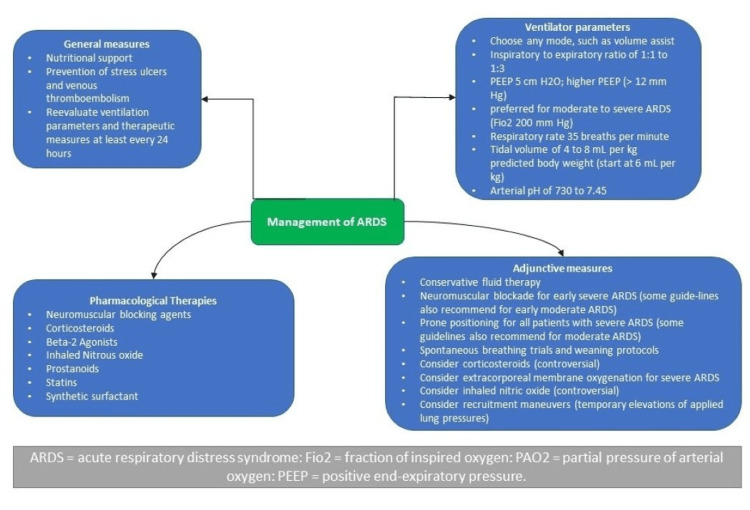
Management of ARDS

The treatment of ARDS typically aims to increase blood oxygen levels, provide breathing support, and treat the underlying cause of the disease. Most ARDS patients are placed on a mechanical ventilator, usually in the ICU. The Faculty of Intensive Care Medicine and Intensive Care Society (FICMICS) Guideline Development Group has used the GRADE methodology to make the following recommendations for managing adult patients with ARDS. In this review article, we aim to discuss the following treatment interventions based on recent guidelines (Figure 2).

Mechanical ventilation at a low tidal volume

The FICMICS group recommends the routine use of lower tidal volumes to manage patients with ARDS (GRADE recommendation: strongly in favor) [13]. The recommendation to use lower tidal volume (less than or equal to 6 mL/kg predicted body weight) ventilation with a plateau pressure less than or equal to 30 cmH_2_O is strong despite the moderate quality of evidence for hospital mortality and barotrauma, but the low quality of evidence for 60-day mortality. The evidence was downgraded for serious indirectness for hospital mortality and inconsistency and imprecision for 60-day mortality. For example, the beneficial effects of low tidal volume ventilation were only seen in one large trial, and the means of managing respiratory acidosis in the ARDS Network ARMA trial is not generally applied. However, a lack of adverse effects associated with the intervention, a strong mechanistic rationale for its use, and supportive data from ARDS prevention studies have resulted in its universal acceptance as a gold standard of care [14-16]. One study showed a significant reduction in the risk of death in the lower tidal volume and higher PEEP group at 28 days (RR: 0.54, 95% CI: 0.31 to 0.91, p=0.02) [17]. Similarly, pooled data from two studies showed a significant reduction in the risk of ICU mortality (RR: 0.57, 95% CI: 0.40 to 0.82, p=0.002) and hospital mortality (RR: 0.62, 95% CI: 0.44 to 0.87, p=0.006) [13].

Positive end-expiratory pressure

FICMICS recommends high PEEP strategies for patients with moderate or severe ARDS (P/F ratio <200 mmHg: GRADE recommendation: weakly in favor) [13].

FICM identified low-quality evidence to support higher PEEP strategies in ventilation patients with moderate or severe ARDS. Evidence was downgraded because of inconsistency caused by differences between individual studies in the strategy to set the PEEP level. Some trials compared lower tidal volume ventilation as part of a ventilator strategy incorporating higher PEEP levels. The recommendation to consider using higher PEEP in patients with at least moderate ARDS is based on subgroup and individual patient data meta-analysis, providing less robust evidence than a randomized clinical trial (RCT) investigating higher PEEP in this patient group. The risk of barotrauma due to the use of higher PEEP for patients with at least moderate or severe ARDS cannot be excluded because this risk has not been quantified in this population. The quality of this evidence is also limited by inconsistency, as the meta-analysis included trials of high PEEP with different tidal volume strategies [11,13]. A higher PEEP ventilation strategy was not associated with increased rates of air leaks (RR: 0.97, 95% CI: 0.66 to 1.42), with the evidence supporting this finding deemed very low because of the difference in tidal volume strategies assessed between the intervention and control arms of some studies, and the imprecision of the results [18].

Extracorporeal membrane oxygenation (ECMO)

FICM does not recommend the routine use of ECMO for all patients with ARDS (GRADE recommendation: weakly against). These guidelines suggest using ECMO with lung-protective mechanical ventilation in selected patients with severe ARDS (GRADE recommendation: weakly in favor) [13,19].

The use of ECMO in selected adults suffering from severe ARDS was given a weakly positive recommendation based on very low-quality evidence. The most widely used indications for ECMO are those reported in the CESAR study [19]. There is a lack of data to make this judgment: one RCT remains after excluding studies including patients supported with ECCO2R and one RCT from 1979 in which mechanical ventilation was not protective. Arguably the predominant mechanism through which ECMO may confer a benefit is by enabling the dramatic reduction of ventilation volumes and pressures, thereby mitigating ventilator-associated lung injury (VALI) [13]. Scant evidence of poor quality suggested an increased risk of bleeding associated with using ECMO: consistent with data from the extracorporeal life support organization (ELSO), which publishes its registry data from around 300 centers worldwide. The incidence of severe bleeding (approximately 15% overall) and intracranial hemorrhage (3.9%) associated with the use of venovenous ECMO for respiratory failure in adult patients based on data from the ELSO registry from its inception in 1989 to 2016 has recently been reported [20]. ECMO is associated with the risk of severe bleeding, although this has not been universally reported or consistently defined in published studies. The risk ratio for bleeding associated with ECMO was 11.44 (3.11-42.06). The quality of evidence was deemed very low because data were based on two non-randomized studies that only included patients with ARDS associated with influenza A (H1N1) [21,22].

Corticosteroids

The use of corticosteroids in established ARDS should be explored by a suitably powered, multicentre RCT with long-term follow-up (GRADE recommendation: research recommendation) [13,23,24]. Current evidence includes the possibility of substantial patient benefit, and the risk of harm appears small. However, the group has noted that the trials did not involve a longer-term follow-up of survivors. The evidence is of low to very low quality from clinical trials mainly conducted before the current era of lung protective ventilation. In addition, the lack of sufficient power in any individual study or the combined meta-analysis as well as the heterogeneity of the dose, timing, and agent used also influenced the decision. The group believed that a position of equipoise exists, and the research recommendation reflects this view. Potential harms of treatment with steroids included excess hospital-acquired infections, ICU-acquired weakness, and delirium. The only meta-analysis that exists reported a composite analysis of infection, ICU-acquired weakness, diabetes, gastrointestinal bleeding, and other complications [25]. A recent study on dexamethasone treatment for ARDS reported that early administration of dexamethasone could reduce the duration of mechanical ventilation and overall mortality in patients with established moderate-to-severe ARDS [26].

As a word of caution, it is worth mentioning that specific steroid-responsive disorders may mimic ARDS, for example, Pneumocystis jiroveci pneumonia, acute eosinophilic pneumonia, and diffuse alveolar hemorrhage [23,24].

Extracorporeal carbon dioxide removal (ECCO_2_R)

The use of ECCO_2_R in established ARDS should be the subject of a suitably powered multicentre RCT with long-term follow-up and economic analysis (GRADE recommendation: research recommendation) [13,27].

Potential harms of treatment with ECCO_2_R included bleeding and thrombosis. Complications depended on the type of ECCO_2_R used with approaches that required arterial cannulation reporting an incidence of arterial injury from 0% to 25% [27]. Blood transfusion requirements were also higher in the ECCO_2_R group [27]. Current evidence is extremely limited and mainly consists of non-randomized prospective and retrospective trials [28-29]. The differences between ECCO_2_R and conventional ventilation techniques make the two RCTs incomparable. However, there is evidence to indicate that ECCO_2_R can allow ventilation with tidal volumes lower than those currently recommended for ARDS, and the potential benefits of this approach should be tested in an appropriately designed RCT. The group believed that a position of equipoise exists, and the research recommendation reflects this view.

Inhaled vasodilators

FICM does not suggest using inhaled nitrous oxide (iNO) in patients with ARDS (GRADE recommendation: weakly against) [13]. The recommendation that iNO not be used for adult patients with ARDS is based on low-quality but consistent evidence suggesting a lack of mortality-related benefit and an association with renal dysfunction [30]. While the studies examining the role of iNO in ARDS are imperfect, further trials would be given a low priority. The possible use of iNO in patients with severe right ventricular dysfunction or extreme hypoxemia for short periods, while more long-term rescue strategies (such as ECMO) are instituted, falls outside the scope of these guidelines [30].

The administration of iNO was associated with an increased incidence of renal dysfunction in four trials representing 80% of the patients recruited into the nine studies analyzed above (RR: 1.50, 1.11 to 2.02). The quality of the evidence supporting the association was judged to be low based on the factors outlined above and the variable criteria used to define renal dysfunction. However, the consistency between trials was good [31,32].

Neuromuscular blocking agents (NMBAs)

FICM does not suggest using NMBAs for all patients with ARDS (GRADE recommendation: weakly against). These guidelines recommend cisatracurium besylate with continuous 48 hours of infusion in patients suffering from early moderate/severe ARDS (P/F <20 kPa: GRADE recommendation: weakly in favor) [13].

The use of cisatracurium besylate in adults suffering from early, severe ARDS was given a weakly positive recommendation based on moderate evidence quality. The group felt that it was appropriate to recommend this management protocol because it was the only one studied by RCT. Due to the nature of this intervention, it should only be given to patients who are adequately sedated and receiving invasive ventilation. It would have been challenging to recruit patients with mild ARDS [33].

A key concern in terms of using NMBA in the ICU is the presumed risk of increased ICU-acquired weakness with their use. Although the risk of ICU-acquired liability was not found to be significantly improved on meta-analyses (RR: 1.08; 95% CI: 0.83 to 1.41), these findings are severely limited by the lack of robust screening measures in two of the contributing RCTs and by the lack of follow-up beyond ICU discharge in the final RCT [34,35]. The recent results from a ROSE trial suggested that among patients with moderate-to-severe ARDS who were treated with a strategy involving a high PEEP, there was no significant difference in mortality at 90 days between patients who received an early and continuous cisatracurium infusion and those who were treated with a usual-care approach with lighter sedation targets [36].

Prone positioning

The FICM group does not recommend using prone positioning for all patients with ARDS. Prone positioning is recommended for a PF ratio <150 mmHg for 16 hours/day in patients with moderate/severe ARDS (P/F ratio <20 kPa: GRADE recommendation: strongly in favor) [13].

Current evidence includes the possibility of substantial patient benefit in terms of reduced mortality when combined with lung-protective ventilation and delivered for at least 12 hours to patients with moderate/severe ARDS. Evidence for these findings was of moderate quality. The relative improvements in study design over the time of publication of all eight trials were such that the most recently published studies focused enrolment on the most severe strata of patients with ARDS and involved a multimodal intervention comprising lung-protective ventilation with prolonged prone positioning producing highly favorable outcomes [37]. This observation provides the rationale for the robust classification of recommendations.

The possibility for substantial patient benefit must be considered in the context of a significant risk of adverse events, including endotracheal tube displacement, pressure sores, and loss of venous access. However, the evidence to support these findings was either low or very low. However, the guidelines development group felt that these adverse events could be mitigated by ensuring that sufficient skilled personnel was in place to deliver and monitor the intervention [38-40]. Overall, the pooled risk of any adverse event with prone positioning was significantly increased (RR: 1.10; 95% CI: 1.01 to 1.12). Where a more detailed analysis of adverse events was conducted, endotracheal tube displacement (RR: 1.33; 95% CI: 1.02 to 1.74), the incidence of pressure sores (RR: 1.23; 95% CI: 1.07 to 1.41), and loss of venous access (RR: 1.98; 95% CI: 1.11 to 3.55) were significantly increased. However, this evidence was downgraded based on the risk of bias and imprecision in the trials evaluated [13].

Discussion

There are no disease-modifying drug therapies for ARDS. Drug development in this area is notoriously difficult, partly because ARDS is not a disease but a syndrome involving acute respiratory failure occurring de novo due to various conditions. One strategy designed to increase the likelihood of positive clinical trials in ARDS is to select a less heterogeneous patient population: a step on the road to a personalized approach made at the expense of having a smaller pool of patients to recruit. Such splitting can be envisaged based on readily identifiable predisposing causes [e.g., influenza pneumonia, transfusion-related acute lung injury (TRALI), or systemic sepsis] or inherent patient characteristics, such as alcoholism or the expression of particular single nucleotide polymorphisms known to be associated with a predisposition to ARDS. The ultimate aim is to identify subgroups, so-called endotypes of ARDS, that predict a positive response to a certain class of therapy [41]. Current management of ARDS is hampered by a failure to diagnose the condition and prevent iatrogenic harm. We need to heighten awareness about the diagnosis, particularly outside the ICU, so that the opportunity to prevent the syndrome's progression is not missed. Research into prevention and treatment needs to be translated more effectively into clinical settings. Biomarkers that confirmed the diagnosis shed light on patients with a poor prognosis and predicted that a positive response to a particular therapy would be invaluable in research and clinical care. For example, a validated bedside biomarker of VALI would facilitate the fine-tuning of mechanical ventilation. It could guide related decision-making during the recovery phase of ARDS, for example, assessing the risk-benefit relationship between allowing spontaneous ventilatory modes with associated larger tidal volumes. To discover effective drug therapies, continued investment in human studies that aim to elucidate the pathogenesis of ARDS is essential to identify clinically useful biomarkers and surrogate outcome measures [42,43]. These investigations need to be performed to design a stepwise approach to testing novel therapeutics in this particularly challenging patient group [44]. Finally, standardization of outcome measures will help conduct and compare clinical trials; such work is underway, and the results will be available soon.

## Conclusions

ARDS is a clinically and biologically heterogeneous disorder associated with various disease processes that lead to acute lung injury, increased non-hydrostatic extravascular lung water, reduced compliance, and severe hypoxemia. Despite significant advances in the field, mortality associated with this syndrome remains high. Mechanical ventilation remains the most important aspect of managing patients with ARDS. In-depth knowledge of lung protective ventilation, optimal PEEP strategies, modes of ventilation, and recruitment maneuvers is essential for the ventilatory management of ARDS. Although the management of ARDS is constantly evolving as new studies are published, guidelines are being updated, and further data is warranted from RCTs for the early and late phases of ARDS, including severe ARDS.
